# Draft Genome Sequence of *Glutamicibacter* sp. Strain JC586, Isolated from Soil Sediment of the Floating Islands of Loktak Lake, India

**DOI:** 10.1128/mra.00746-22

**Published:** 2022-09-20

**Authors:** Khongsai L. Lhingjakim, Chintalapati Sasikala, Chintalapati Venkata Ramana

**Affiliations:** a Department of Plant Sciences, School of Life Sciences, University of Hyderabad, P.O. Central University, Hyderabad, India; b Bacterial Discovery Laboratory, Centre for Environment, IST, JNT University Hyderabad, Kukatpally, Hyderabad, India; University of Delaware

## Abstract

The 3.52-Mbp whole-genome sequence of a *Glutamicibacter* sp. strain isolated from soil sediment of the floating islands of Loktak Lake is reported. The genomic information here gives insight into the presence of genes linked to oxidative stress, osmo-protection, and cold shock proteins which helps in the survival of the organism under extreme environmental conditions.

## ANNOUNCEMENT

The genus *Glutamicibacter* belongs to the phylum *Actinomycetota*, consisting of Gram-positive, high GC, rod-coccus cells. Although members of *Glutamicibacter* have been isolated from different ecological niches, relatively little is known about their physical attributes that allow these organisms to survive in harsh environments (1, 2).

*Glutamicibacter* sp. strain JC586 was isolated from a soil sample collected from the floating islands or Phumdis (3) of Loktak Lake, India (24°30′21″ N/93°47′43″ E). Phumdis constitute a dense rhizosphere extending down to the sediment of the lake and hence serve as an ecological habitat for several groups of bacteria (4). The lake is an ecological hotspot with a remarkable diversity of flora and fauna and was declared a Ramsar site (a wetland of international significance) in 1990. Briefly, a serially diluted rhizosphere soil sample was plated onto nutrient agar and incubated at 30°C for 7 days. Yellow-colored colonies were selected from the 10^−4^ dilution which were further purified by repeated streaking. Genomic DNA was isolated from a single colony using the Nucleo-pore genomic DNA (gDNA) fungal bacterial mini kit from Genetix Biotech Asia Pvt. Ltd., India, and the genome sequence was outsourced to AgriGenome Pvt. Ltd. (Kochi, India). Library preparation was carried out using the NEBNext Ultra DNA library preparation kit. The Illumina HiSeq 2500 instrument was used for whole-genome sequencing. The total number of raw reads generated was 4,730,390 (forward and reverse strands) with a read length of 100 bp. The fastq files were trimmed by removing the adapter sequences using Cutadapt version 1.11 (5) and by filtering out reads with an average quality score Q of <30 in any of the paired-end reads using Sickle version 1.33 (6). The unique reads were fetched using FastUniq version 1.1 (7), and finally, plasmids were removed from the cleaned reads using Bowtie2 version 2.2.6. *De novo* assembly was performed using ABySS version 2.0.2 with a *k*-mer range of 31 to 95 (8). Default parameters were used for all software unless otherwise specified. The draft genome of *Glutamicibacter* sp. JC586 contains 28 contigs, with 3,524,842 bp, and the *N*_50_ value was 558,740 bp with 100× coverage and a GC content of 60.04%. Genes were predicted using the NCBI Prokaryotic Genome Annotation Pipeline (PGAP) version 5.0 which shows 3,294 total predicted genes; 3,177 coding genes; and 75 RNAs, including 62 tRNAs, 10 rRNAs, and 3 noncoding RNAs (ncRNAs) (Table 1).

**TABLE 1:** Genome assembly statistics and GenBank accession numbers of *Glutamicibacter* sp. JC586

Metric	Data
Source	Soil
Sampling date (day/mo/yr)	28/12/2017
Sampling location	24°30′21″ N, 93°47′43″ E; Loktak Lake, India
Genome size (bp)	3,524,842
GC content (%)	60.4
No. of contigs	28
*N*_50_ (bp)	558,740
*L*_50_ (bp)	3
No. of rRNAs	10
No. of tRNAs	62
No. of genes (coding)	3,177
No. of pseudogenes	42
BioSample accession no.	SAMN12096126
GenBank WGS^*a*^ accession no.	NZ_VHIN00000000
GenBank assembly accession no.	GCF_009805585.1

a WGS, whole-genome sequencing.

An EzBioCloud BLAST analysis of the 16S rRNA gene sequence (1,514 nucleotides [nt]) of strain JC586 yielded the highest identity (99.3%) with the type strain of Glutamicibacter halophytocola KLBMP 5180, with an overall average nucleotide identity (ANI) (9) value of 79.53% and DNA-DNA hybridization (dDDH) (10) of 21%. Annotation of the JC586 genome reveals several genes related to ecological adaptation. They include genes putatively encoding oxidative stress responses (*sodA*, *soxR*, *trxB*, *ohr*, and *ahpC*), osmo-protectants (*otsB*, *glgB*, *glgC*, *glgP*, and *prop*), and cold shock response (*infA*, *infB*, *infC*, *rbfA*, and *nusA*) (11).

### Data availability.

The whole-genome sequence of *Glutamicibacter* sp. JC586 has been deposited at GenBank under the accession number NZVHIN00000000, the BioProject accession number PRJNA224116, and BioSample accession number SAMN12096126. The raw sequences have been deposited in GenBank under the SRA accession number SRR19316923. *Glutamicibacter* sp. JC586 has been deposited in Biological Resource Center, NITE (NBRC), and Korean Agricultural Culture Collection (KACC) under the accession numbers 113657 and 21453, respectively.
